# Gender Differences in Body Satisfaction Perception: The Role of Nutritional Habits, Psychological Traits, and Physical Activity in a Strength-Training Population

**DOI:** 10.3390/nu16010104

**Published:** 2023-12-28

**Authors:** Jorge Jimenez-Morcillo, Vicente Javier Clemente-Suárez

**Affiliations:** 1 Faculty of Sports Sciences, Universidad Europea de Madrid, Tajo Street, s/n, 28670 Madrid, Spain; jorge.jimenez2@universidadeuropea.es; 2 Grupo de Investigación en Cultura, Educación y Sociedad, Universidad de la Costa, Barranquilla 080002, Colombia

**Keywords:** body satisfaction, gender differences, nutritional habits, psychological profiles, physical activity, societal norms

## Abstract

The objective of this study is to examine gender disparities in body satisfaction perception, emphasizing the influence of nutritional habits, psychometric assessments, levels of physical activity, and health-related metrics. Employing a sample of 605 strength-trained participants (385 males and 224 females), aged between 20 and 35 years, and regularly engaged in strength training, we conducted a thorough analysis using Google Forms. We evaluated variables including age, anthropometric data, resistance training frequency, food consumption patterns, and psychological profiles. Our findings indicate significant gender-based differences in body satisfaction perception. Females tend to overestimate their muscular size and express heightened concern regarding gluteal shape, while males exhibit a more realistic self-perception, primarily focused on hip width. Dietary patterns also display gender-specific tendencies; females prefer healthier options like vegetables, whereas males consume more milk, fermented products, and carbohydrates. Hydration practices diverge as well, with females showing higher water intake in contrast to males’ preference for alcoholic and carbonated beverages. Psychologically, males demonstrate greater extroversion, while females exhibit higher conscientiousness, openness, and a tendency toward negative thoughts and anxiety. Regarding physical activity, females engage in training sessions with greater volume compared to males. This study highlights the intricate interplay of social, cultural, and personal factors shaping gender-specific perceptions of body satisfaction and their subsequent impact on health and lifestyle choices. These insights pave the way for future specialized interventions and research, underscoring the importance of understanding gender-specific nuances to promote healthy body satisfaction and self-perception.

## 1. Introduction

In recent years, there has been a significant increase in the number of people using fitness centers and engaging in physical exercise for both aesthetic and health reasons [1]. Regular exercise is associated with a reduced risk of chronic diseases, such as type 2 diabetes, cardiovascular disease, and some types of cancer [2]. Moreover, the desire to achieve a fit body has become a cultural trend, as indicated by the growing popularity of social media influencers and fitness models [3]. This trend underscores the need for a deeper understanding of body satisfaction perception and its broader implications for strength-trained subjects. The concern for physical appearance and aesthetics is a phenomenon that is increasingly common today. In fact, dissatisfaction with physical appearance and the pursuit of aesthetic perfection can have serious consequences for individuals’ health [4]. Body satisfaction plays a crucial role in mental health, contributing to self-esteem and overall well-being [5]. Dissatisfaction with physical appearance, on the other hand, is linked to negative mental health outcomes, including depression, anxiety, and eating disorders [6]. The pursuit of aesthetic perfection, fuelled by societal and media influences, can lead to unhealthy behaviors like excessive exercise or disordered eating [7]. Therefore, promoting a balanced and realistic body image is essential for both physical and mental health in strength-trained subjects [5].

The way people perceive their own bodies is strongly influenced by cultural and societal norms, as well as personal experiences. Negative body satisfaction, which involves a distorted and dissatisfied perception of one’s own body, is associated with a variety of negative consequences. including low self-esteem, depression, and disordered eating behaviors [5]. Individuals with eating disorders, such as anorexia nervosa and bulimia nervosa, often have a distorted body satisfaction that leads them to engage in extreme weight loss behaviors, such as restrictive eating. Purging and over-exercising [6] highlight the importance of exploring these perceptions. Our study investigates the nuances of body satisfaction among different genders among strength-trained subjects. In addition, studies have found that poor body satisfaction is a risk factor for the development of disordered eating behaviors, particularly among strength-trained young women [7].

The relationship between body satisfaction and physical activity is complex and bidirectional. Strength-trained individuals who have positive body satisfaction are more likely to engage in physical activity and maintain an active lifestyle [8]. Conversely, regular physical activity can improve body satisfaction and self-esteem, particularly in individuals who engage in exercise for weight control [9]. However, body dissatisfaction and negative body satisfaction can also discourage strength-trained individuals from participating in physical activity, particularly in public or group settings [10]. This bidirectionality forms a crucial aspect of our study’s focus. Therefore, interventions that address body satisfaction concerns may be beneficial in promoting physical activity and improving overall health and well-being [6].

Gender-specific nuances in body satisfaction perception form the cornerstone of our research. Research has consistently shown that strength-trained women tend to report more negative body satisfaction than strength-trained men, with higher levels of body dissatisfaction, appearance anxiety, and disordered eating behaviors. This gender difference may be partly explained by the greater emphasis placed on appearance and thinness in female socialization and media representations, as well as by the different sociocultural norms and expectations regarding body size and shape for men and women [11].

In contrast, strength-trained men are not immune to societal pressures concerning body satisfaction. While the idealized male physique has traditionally been associated with muscularity and strength, recent studies have revealed a growing emphasis on muscularity and leanness, indicating a shift in male body ideals [12]. Strength-trained men may encounter distinct challenges and experience body dissatisfaction when they perceive their bodies as falling short of these muscular ideals, potentially leading to behaviors such as excessive exercise or the utilization of anabolic steroids to enhance their physical appearance [13].

To thoroughly investigate gender disparities in body satisfaction, our study delves into the distinct perspectives and concerns of different genders. For strength-trained women, the primary focus of body satisfaction often aligns with societal standards of thinness and beauty, as evidenced by extensive research on the subject [5]. In contrast, strength-trained men’s body satisfaction increasingly gravitates towards muscularity, reflecting a different set of societal expectations and norms [6]. Recognizing and dissecting these gender-specific concerns is essential. It allows for the development of targeted interventions and societal reforms aimed at fostering healthier body satisfaction and self-perception for individuals of all genders, thereby addressing the unique challenges each gender faces [14].

The primary objectives of this study were to examine disparities in body satisfaction perception between strength-trained males and females’ perceptions based on a range of factors, including nutritional habits, psychometric measurements, physical activity levels, and health-related metrics. Specifically, we seek to elucidate the extent and nature of these disparities, focusing on the different ways strength-trained men and women perceive and react to body satisfaction pressures. We aimed to assess whether there are gender differences in the perception of body satisfaction, with a specific focus on strength-trained women’s heightened vigilance toward their own body satisfaction as influenced predominantly by their nutritional consumption habits and specific psychological profile-related behaviors.

Our study is structured to systematically explore how these gender differences in body satisfaction perception, shaped by cultural, social, and personal factors, manifest distinctly in strength-trained men and women in terms of their nutritional habits and mental health-related behaviors. It is posited that strength-trained women may exhibit a greater tendency towards body dissatisfaction linked to socially promoted thinness ideals, impacting their dietary habits and psychological behaviors. Conversely, strength-trained men may experience dissatisfaction primarily related to musculature and strength, influencing their physical exercise practices and the adoption of extreme measures for muscle development. This investigation is pivotal in contributing to a more nuanced understanding of gender-specific body satisfaction perceptions and their impact on health and lifestyle choices.

## 2. Materials and Methods

In the current study, a total of 605 participants, of whom 385 are male and 224 are female, all engaged in strength training, were interviewed online. During the registration process, survey respondents were required to provide their full names and email addresses. These pieces of information were treated as strictly confidential and handled in accordance with data privacy regulations.

Study participants were invited to complete the survey by accessing a dedicated Google Forms link or scanning a provided QR code. Prior to their engagement in this study, all participants received comprehensive information concerning this research objectives and procedures. The inclusion criteria for this study required that both male and female participants be within the age range of 20 to 35 years and have engaged in strength training with a weekly frequency of 2 to 7 days, continuously for at least 6 months. To ensure the reliability of responses, each participant’s submission was verified for consistency and completeness. Moreover, potential biases in responses were assessed through a preliminary analysis, identifying any patterns of skewed or unusual replies. It was explicitly communicated that their participation was entirely voluntary, and they retained the option to withdraw at any point during the study without incurring any penalties or consequences. “In order to participate, individuals were required to provide their digital consent by signing an informed consent form, thereby indicating their full understanding and willingness to participate in this research”.

This research adhered to the principles outlined in the Helsinki Declaration (revised in Brazil in 2013) regarding human research ethics. Ethical approval for the study was obtained from the University Ethics Committee (CIPI/18/074), ensuring that this research was conducted in accordance with established ethical guidelines.

Through the Google Forms questionnaire. The following variables were analyzed: 

*Age and anthropometric variables*: Gender, age (years), height (cm), weight (kg), and body mass index (BMI. kg/m^2^) were obtained.

*Resistance training frequency and intensity*: These parameters were evaluated by quantifying weekly training sessions, categorized into aerobic and strength training: The volume of aerobic exercise was measured in minutes of weekly training to quantify strength training intensity. Subjects were asked what percentage of their weekly training was below 50% of their one-repetition maximum (1RM). Between 50% and 70%, between 70% and 85%, and above 85%. Lastly, percentages of 1RM for the back squat, deadlift, and bench press were also recorded.

*Food consumption variables*: We analyzed a food consumption frequency questionnaire that inquired about the frequency and quantities of various food items (juices 250 mL, water glasses 250 mL, alcoholic beverages 250 mL, fermented beverages 250 mL, soft drinks 250 mL, energy drinks 250 mL, milk glasses 250 mL, fruits 90 g, bakery/sweets 90 g, meat 150 g, fish 150 g, legumes 200 g, pasta or rice 150 g, vegetables 200 g, bread 50 g, fast food 180 g, whole foods 150 g, gel consumption 40 g, muesli bars 150 g, and protein drinks 300 mL). For this purpose, we utilized the European Prospective Investigation into Cancer and Nutrition Food Frequency Questionnaire (EPIC-FFQ), specifically adapted and validated for Spanish populations [15,16].

*Nutritional habits*: In terms of nutritional habits, measurements were taken for the number of takeout meals, the frequency of dining out, and the days when meals were prepared at home.

*Psychological profile*: The psychological profile was assessed using various scales and inventories. The Spanish version of the Big Five Inventory [17], with an alpha coefficient of 0.73, was employed to measure the five personality traits: openness to experience. conscientiousness. Extraversion, agreeableness, and neuroticism The reduced version of this inventory consisted of 44 items, with responses rated on a 5-point Likert scale ranging from 1 (completely disagree) to 5 (completely agree).

Anxiety levels were evaluated using the reduced version of the Spanish adaptation of the Spielberger State-Trait Anxiety Inventory, which had an alpha coefficient of 0.93 [18]. This version included 6 items assessing anxiety, with responses rated on a 4-point Likert scale ranging from 1 (not at all) to 4 (very much).

The Spanish version of the Acceptance and Action Questionnaire II [19] was utilized to measure experiential avoidance or psychological inflexibility. This questionnaire consisted of 7 items, with responses rated on a 7-point Likert scale ranging from 0 (never) to 7 (always). The alpha coefficient for this scale was 0.84 (ranging from 0.78 to 0.88).

Loneliness was measured using the Spanish version of the UCLA Loneliness Scale, which had an alpha coefficient of 0.94 [20]. The condensed version of this scale included 3 items, with responses rated on a 3-point Likert scale ranging from 1 (never) to 3 (frequently).

The Spanish version of the ZUNG Depression Scale [21], specifically adapted for assessing depression in the context of the COVID-19 crisis, was utilized. This self-report scale consisted of 20 items formulated in positive and negative terms. It demonstrated an alpha coefficient of 0.09 and had a high sensitivity and specificity of over 80%. The scale encompassed eight items each for somatic and cognitive symptoms, along with two items for mood and two for psychomotor symptoms.

To set differences in body satisfaction perception between strength-trained men and women, the following variables were measured using the eating disorder inventory [22], based on a numeric scale ranging from 1 (never) to 6 (always): satisfaction level with stomach size, muscle size, silhouette, buttock shape, thigh and hip size, body satisfaction, and body weight. Additionally, a scale ranging from 1 to 9 was employed, featuring figures representing different body silhouettes, with 1 being the thinnest and 9 the most voluminous. On this scale, subjects indicated which figure they identified with and which one they considered ideal.

### Statistical Analysis

The authors used SPSS version 24.0 (SPSS Inc., Chicago, IL, USA) to conduct their statistical analyses. Descriptive statistics, such as means and standard deviations, were calculated for each variable. The normality and homogeneity of each variable were assessed using Kolmogorov-Smirnov tests. An independent *t*-test was used to analyze the differences in nutrition in the sociodemographic, academic, and psychological variables. The authors set the significance level at *p* ≤ 0.05.

## 3. Results

In the analysis of the obtained results, substantial disparities in participant demographics came to light. Notably, it was evident that strength-trained female participants exhibited a statistically significant higher mean age in comparison to their strength-trained male counterparts. Furthermore, a marked contrast in body weight was observed, with strength-trained male participants displaying a statistically significant higher mean body weight (Table 1).

Regarding nutritional data, it was observed that strength-trained men were more involved in food preparation compared to strength-trained women. Additionally, strength-trained women consumed a higher number of glasses of water daily than strength-trained men. Men, on the other hand, were found to consume more alcohol weekly, along with an increased consumption of soda. Similarly, men reported higher weekly consumption of glasses of milk, fermented foods, pastry sweets, cheese, and eggs compared to women. In terms of weekly food consumption, men reported higher consumption of processed meat, rice, pasta, bread, fast food, gels, and cereal bars compared to women. However, women reported consuming more cooked vegetables (Table 2).

In the context of variables influencing body satisfaction, several notable differences were observed. Men tended to perceive their muscle size as larger compared to women, whereas men exhibited a more objective self-assessment of their muscle size. Conversely, men perceived their hip width as wider than women. Furthermore, women expressed more concern about the size of their buttocks, considering it more significant than men did. Additionally, women exhibited a more favorable perception of others’ silhouettes, engaging in fewer self-comparisons with others. In terms of psychological profiles, men demonstrated a higher degree of extroversion compared to women. Women, on the other hand, exhibited higher levels of conscientiousness and a greater openness to new experiences. Women reported a higher frequency of negative thoughts and displayed less effective coping mechanisms in response to stress. According to the ZUNG Score results, women showed a greater number of symptoms associated with depressive behaviors than men, and they also presented higher levels of anxiety compared to men (Table 3).

Regarding health parameters, significant distinctions emerged. Men reported a higher frequency of experiencing a dry throat compared to women. Additionally, over the past year, men reported a longer duration of illness than women. Concerning weekly training intensity, it was noted that women engaged in training at a higher volume, below 50% intensity, compared to men (Table 4).

## 4. Discussion

Our study investigated gender-related disparities in body satisfaction perception, shedding light on distinct patterns among strength-trained participants. Notable is the finding of substantial differences in demographic variables such as age and body weight, where female participants exhibited a higher mean age and lower body weight compared to males, as shown in Table 1. This may contribute to the observed differences in body satisfaction. Our results indicate that strength-trained men tend to perceive their hip width as wider than that of strength-trained women. In terms of size satisfaction, men showed more muscular size satisfaction compared to women, a phenomenon that can be attributed to the weight lifted in bench press and back squat exercises. This are consistent with the reported higher muscle mass and strength in men, as reflected in Table 4’s data on bench press and back squat personal records. Our findings are consistent with those reported in the literature [23]. Furthermore, this study revealed that strength-trained women demonstrated increased concern regarding the size of their buttocks, deeming it to be of greater significance compared to the perception held by strength-trained men. This aligns with the nutritional data in Table 2, where women’s dietary choices, like higher vegetable consumption, could be influenced by their body satisfaction concerns. Importantly, our findings also revealed that women exhibited a more favorable perception of others’ silhouettes when compared to men, who engaged in fewer self-comparisons with their peers. This might be influenced by the psychological profiles presented in Table 3, where women showed higher levels of conscientiousness and openness to new experiences. These comprehensive insights contribute significantly to our understanding of how gender influences body satisfaction perception, offering valuable implications for research in psychology and the health sciences.

The initial hypothesis of our study contended that gender differences in body satisfaction are profoundly influenced by societal norms [24], which in turn significantly affect nutritional habits and psychological profiles. The significant differences in nutritional habits between genders, as shown in Table 2, support this hypothesis. Men’s greater involvement in food preparation and higher consumption of processed foods and alcohol could reflect societal influences on dietary choices. We posited that women modify their diet to pursue a slim physique, while men tailor their eating habits towards achieving enhanced strength and muscle mass. Our findings corroborate that body satisfaction disparities are indeed conditioned by the pursuit of specific gender-based stereotypes driven by social and cultural influences. This is evidenced by the differences in dietary patterns, where men’s higher consumption of carbohydrates and women’s preference for vegetables (Table 2) reflect these societal norms [25]. However, this study also reveals that the psychological behaviors and nutritional practices undertaken to achieve these gender stereotypes challenge the prevailing dogma. Particularly, the higher levels of anxiety and depressive behaviors in women, as indicated by the ZUNG Score in Table 3, suggest a complex interplay between psychological factors and body satisfaction. This breakthrough provides novel avenues for research in the realm of body diversity, offering fresh perspectives on the distinctions and commonalities in body satisfaction among women and men.

In addition, the health parameters in Table 4, such as the longer duration of illness reported by men, may have implications for body satisfaction and perceived health. This could be influenced by their dietary and lifestyle choices, as indicated by their higher consumption of alcohol and processed foods. These distinctions align with prior findings in existing literature [26]. Such distinctions may offer perspective on the extended life expectancy observed among women. The contrast in hydration practices, with women showing a higher water intake, could be a contributing factor to this extended life expectancy. The contrast in hydration practices, with women showing a higher water intake, could be a contributing factor to this extended life expectancy. Our study delineates a contrast in hydration practices: women exhibit higher water intake, while men, conversely, tend toward excessive consumption of carbonated beverages and alcoholic drinks. Several scholars have proposed the presence of a gender disparity associated with these patterns of alcohol intake [27], suggesting a potential reason for the increased occurrence of renal diseases in men compared to women [28]. The detailed nutritional data in Table 2 corroborates these patterns, showing men’s higher consumption of soda and alcohol. Regarding dietary patterns, consistent with prior research [29], our findings suggest that women tend to adhere to a significantly healthier dietary regimen compared to men, showing a preference for vegetable consumption over other food categories. This is further supported by the nutritional data in Table 2, where men reported higher consumption of processed meat, rice, pasta, bread, fast food, gels, and cereal bars compared to women. Further studies support this trend, indicating that women favor other healthful food categories, such as fruits, in comparison to men [30]. Additionally, our research findings illustrate that men tend to consume more carbohydrate-rich foods, including rice, pasta, confectioneries, and dairy products. This aligns with established literature that highlights the increased consumption of carbohydrates. [31] and dairy products [32] have been linked to ailments like type II diabetes and specific cancer types. In addition, other studies demonstrate that high consumption of dairy products and refined carbohydrates is associated with factors that can lead to metabolic syndrome, such as obesity and insulin resistance [33]. This disparity in dietary habits may elucidate the substantial contrast observed in the prevalence of metabolic syndrome between men and women, as proposed by certain researchers [34].

Certain studies suggest that [35], within the psychological domain, women tend to display greater extroversion during interpersonal interactions compared to men. This is reflected in our findings, where women demonstrated higher levels of conscientiousness and openness to new experiences (Table 3). Moreover, some researchers emphasize that, due to variations in the nervous system’s composition, women are more adept at embracing novel experiences [36] and have access to a higher level of consciousness [37]. Our results support this assertion, showing that women in our study exhibited a greater degree of extroversion and openness to new experiences compared to men (Table 3). The cerebellum is larger in men than in women [38]. This difference, according to some authors, indicates that it is men who have better control over the levels of consciousness [39], although there is currently controversy in this aspect [40]. This information from previous research aligns with our study’s outcomes, illustrating notable dissimilarities in how men and women engage in social interactions, with men exhibiting greater ease. Additionally, prior studies have suggested that interactions vary based on gender configurations, concluding that same-gender interactions may be comparatively more straightforward [41]. Our results indicate that regardless of which gender the subject interacts with, men have an easier time. Regarding stress, there is no clear consensus on which gender is more effective in managing it [42]; however, the results of our study show a significant difference, indicating that women handle stress worse than men and also have a greater predisposition to negative thoughts, in line with the scientific literature [43]. This aligns with the findings of [44], which show that women manage stress worse due to their higher exposure to negative thoughts. Likewise, other researchers have observed that women exhibit elevated levels of anxiety compared to men [45]. However, other studies show the opposite, demonstrating that men have a lower tolerance for anxiety [46]. Based on the outcomes derived from our ZUNG Score, it was evident that women exhibit higher levels of anxiety compared to men. Nevertheless, a correlation was observed between increased exposure to negative thoughts and reduced levels of stress and anxiety tolerance [47]. Men and women employ distinct neural resources in their management of stress and anxiety. These resources are linked to the activity of specific brain regions, including the dorsomedial prefrontal cortex, cerebellum, left parietal, temporal lobes, and occipital gyrus [48]. Nevertheless, other authors attribute these differences in stress and anxiety management to interindividual factors that go beyond merely belonging to one gender or another, such as external stressors, life expectations, family context, social support, or even learning based on behavioral imitation with other individuals [49].

In terms of body satisfaction, our findings suggest significant gender-based differences. Women perceive their muscle size as larger compared to men, which might be influenced by their nutritional and health concerns, as reflected in their higher consumption of cooked vegetables and lower intake of processed meats (Table 1 and Table 2). Additionally, women are more aware of the size of their buttocks and demonstrate better emotional management regarding this aspect than men. This greater emotional control might also be related to the higher levels of consciousness and stress tolerance observed in women (Table 3). Furthermore, women exhibit greater tolerance for comparing themselves with other figures, whereas men show heightened sensitivity when making such comparisons. Extending these observations, other authors [50] link differences in body satisfaction perception to conditions like eating disorders or psychiatric patterns. In agreement with these observations, some studies [51] emphasize the importance of educating on the emotional management of body satisfaction perception to prevent dissatisfaction. Such dissatisfaction could lead to diseases associated with being overweight, including cancer or diabetes. According to our results, women exhibit better emotional management and, consequently, a lower predisposition to overweight-related disorders. Lastly, several studies [52] suggest that other cultural media factors drive behaviors leading to differences in body satisfaction between men and women. For instance, mass media and the idealization of a specific body type impact men more than women.

Regarding health parameters, some authors suggest that men experience more illnesses than women [53]. Our findings support this conclusion, showing that men tend to experience a longer duration of illness annually compared to women, which could be related to their dietary patterns and hydration practices (Table 4). However, alternative perspectives within existing literature emphasize a greater incidence of illness among women in contrast to men. This observation is attributed to three main factors: the extended longevity of women [54], which heightens the probability of higher illness rates; poorer mental health conditions [55]; and a more pronounced impact of cardiovascular diseases [56]. In contrast, other authors suggest that the prevalence of diseases among men and women is not straightforward and can be attributed to genetic, epigenetic, epidemiological, and geographical factors, as well as hormonal differences, all of which are interconnected with the social construct related to gender [57].

In relation to physical activity, the existing literature is not precise in elucidating gender differences in terms of training parameters. Some authors assert that women exhibit greater fatigue resistance than men [58], while others solely allude to the disparity in the utilization of energy substrates [59]. Our study goes further, establishing clear differences in how women train compared to men. The results of our study demonstrate that women train at a lower intensity than men. However, they tend to tolerate higher exercise volumes more effectively. It is noteworthy that other authors attribute gender-related differences in physical activity to differing objectives. On one hand, women aim for caloric reduction and a leaner silhouette, while on the other, men pursue greater muscle mass gain [60]. Apart from physiological variances, these differences align with the findings of our study. Lower-intensity, aerobic-oriented training aligns with the popular belief of ‘weight loss’, while high-intensity training is associated with strength gain. Nevertheless, these assertions have been extensively refuted by the scientific literature [61].

Our study’s findings hold significant practical implications in public health, psychology, and nutrition. Understanding gender differences in body satisfaction perception can inform the design of tailored interventions to promote positive body satisfaction and healthy eating habits. For instance, educational programs focusing on emotional management and body satisfaction perception could be particularly beneficial for women, while campaigns to reduce the consumption of processed foods and carbonated beverages might have a more significant impact on men.

Our study presents limitations in analyzing the impact that these differences in body satisfaction perception have on specific psychological behaviors and particular nutritional habits. Furthermore, our research encounters constraints in effectively correlating our psychological, nutritional, and body image outcomes with the influences emanating from established societal and cultural norms. This limitation underscores the difficulty in drawing definitive links between individual perceptions and actions and the wider social and cultural frameworks. A future line of research could review the underlying causes determining these differences in body satisfaction perception, further exploring the complex interplay between individual and societal factors.

## 5. Conclusions

Our comprehensive investigation in a strength-trained population has unveiled significant gender disparities across various domains, encompassing body satisfaction, dietary patterns, hydration behaviors, health outcomes, psychological attributes, and levels of physical activity, aligning with established scientific literature [62]. Our observations indicate that females tend to overestimate their muscle size and exhibit heightened concern regarding the shape of their gluteal region, in contrast to males, who tend to possess a more realistic self-perception and a heightened focus on hip width, as substantiated by prior researchers [63]. Notably, women exhibit exceptional resilience in self-comparisons and excel in the emotional management of body satisfaction concerns, findings corroborated by previous studies [64].

Within the realm of dietary practices, our results suggest that women tend to favor healthier dietary choices, including increased consumption of vegetables, whereas men display a proclivity for higher intakes of milk, fermented products, pastries, cheese, and eggs. These dietary preferences concur with previous research findings [65]. With respect to hydration behaviors, our investigation reveals elevated water intake among women, while men display a preference for alcoholic and carbonated beverages. Additionally, men report longer durations of illness per year and a higher frequency of dry throat issues, consistent with earlier studies [66,67].

Psychologically, our study demonstrates that men exhibit greater extroversion, whereas women display higher levels of conscientiousness and openness to new experiences, consistent with the conclusions of prior research [68,69]. Nevertheless, women also experience more frequent negative thoughts, elevated anxiety levels, and a heightened predisposition toward depression. Our study contributes additional depth to this topic compared to earlier research, which primarily focused on the increased propensity for depression among women compared to men [70].

Lastly, in accordance with earlier research findings [71], women engage in training sessions of higher volume compared to men in the realm of physical activity. This comprehensive gender-specific analysis offers invaluable insights into the intricate interplay among physical, nutritional, and psychological factors in both genders, providing a solid foundation for future specialized interventions and studies in the field of nutritional science and health.

## Figures and Tables

**Table 1:** Demographic, education, and employment data.

					95% Confidence Interval of the Difference	
Variable	Male	Female	T	*p*	Lower	Upper
Age (Years)	25.2 ± 8.6	27.4 ± 9.9	2.895	0.004	−3.79836	−0.72771
Heigh (cm)	145 ± 173.7	171.5 ± 19.0	1.573	0.116	−0.54486	4.92748
Weight (Kg)	76.3 ± 9.3	61.08 ± 10.4	18.241	0.000	13.61358	16.89863
Body Mass Index (Kg/m^2^)	31.1 ± 3.5	21.8 ± 3.3	0.930	0.353	−8.73810	24.45937

**Table 2:** Nutritional data.

					95% Confidence Interval of the Difference	
Variable	Male	Female	T	*p*	Lower	Upper
Days eating out of home (weekly)	1.5 ± 1.3	1.3 ± 1.4	1.490	0.137	−0.05734	0.41794
Days of ordering takeout (weekly)	6.2 ± 5.3	5.3 ± 7.1	2.583	0.010	0.04555	0.33438
Cook most days (weekly)	1.7 ± 1.6	1.6 ± 0.9	1.896	0.058	−0.00568	0.32474
Satisfaction with the weigh	2.0 ± 0.8	2.1 ± 0.7	1.856	0.064	−0.25946	0.00736
Daily water glasses (250 mL)	3.4 ± 1.8	3.8 ± 1.8	2.267	0.024	−0.66028	−0.04726
Fruit juice consumption (mL) (weekly) (250 mL)	2.4 ± 0.9	2.3 ± 0.9	0.113	0.910	−0.15337	0.17201
Alcohol glass consumption (250 mL) (weekly)	2.6 ± 0.7	2.5 ± 0.6	0.742	0.458	−0.07478	0.16566
Beer consumption (25 mL) (weekly)	2.4 ± 0.8	2.4 ± 0.9	0.466	0.641	−0.11034	0.17897
Alcohol cup consumption (250 mL) (Weekly)	2.6 ± 0.7	2.5 ± 0.6	3.586	0.000	0.15199	0.52000
Cola/Soda consumption (250 mL) (weekly)	2.4 ± 0.8	2.4 ± 0.9	2.306	0.021	0.03481	0.43497
Energy drink consumption (250 mL) (weekly)	2.5 ± 1.0	2.1 ± 1.1	0.416	0.678	−0.24335	0.15833
Milk glasses consumption (250 mL) (weekly)	3.1 ± 2.3	2.7 ± 2.0	2.436	0.015	0.09129	0.85067
Fermented dairy consumption (125 g) (Weekly)	2.8 ± 2.0	2.5 ± 1.7	2.035	0.042	0.01185	0.66548
Sweets/bakery consumption (90 g) (weekly)	2.3 ± 1.2	2.0 ± 1.1	3.146	0.002	0.12283	0.53084
Cheese consumption (5 g) (Weekly)	2.6 ± 1.7	2.1 ± 1.5	3.589	0.000	0.23172	0.79168
Egg consumption (Unity) (Weekly)	2.9 ± 2.4	2.5 ± 2.1	2.094	0.037	0.02595	0.81218
Meat consumption (150 g) (weekly)	3.0 ± 2.3	2.6 ± 2.1	2.194	0.029	0.04571	0.82665
Fish consumption (150 g) (weekly)	2.5 ± 1.7	2.3 ± 1.5	1.467	0.143	−0.07302	0.50439
Processed meat consumption (150 g) (weekly)	2.5 ± 1.6	2.2 ± 1.3	2.080	0.038	0.01594	0.55345
Legume consumption (200 g) (weekly)	2.3 ± 1.4	2.2 ± 1.2	0.560	0.576	−0.16993	0.30552
Rice consumption (80 g) (Weekly)	2.7 ± 2.1	2.3 ± 1.8	2.251	0.025	0.05079	0.74460
Weekly pasta (150 g) consumption	2.6 ± 2.0	2.2 ± 1.5	2.350	0.019	0.06221	0.69536
Weekly fruit consumption (90 g)	3.2 ± 2.5	3.5 ± 2.9	1.179	0.239	−0.73452	0.18337
Weekly raw vegetable (200 g) consumption	2.8 ± 2.2	3.0 ± 2.5	1.177	0.240	−0.63642	0.15955
Weekly cooked vegetable (200 g) consumption	2.8 ± 2.2	3.2 ± 2.6	1.956	0.051	−0.80284	0.00159
Weekly bread (50 g) consumption	3.0 ± 2.2	2.5 ± 1.8	2.628	0.009	0.12098	0.83602
Weekly whole food (150 g) consumption	2.8 ± 2.2	2.5 ± 2.0	1.553	0.121	−0.07497	0.64177
Weekly fast food (180 g) consumption	2.2 ± 1.0	2.1 ± 0.9	2.209	0.028	0.02115	0.36068
Weekly protein drink (300 mL) consumption	2.5 ± 1.4	2.5 ± 1.7	0.286	0.775	−0.22643	0.30364
Weekly gel consumption (40 g)	2.5 ± 1.0	2.3 ± 1.2	2.125	0.034	0.01589	0.40373
Weekly muesli bar (150 g) consumption	2.4 ± 1.1	2.2 ± 1.2	1.905	0.057	−0.00584	0.38625

Unity means one portion. A serving means one serving of a meal. The frequency with which each food/drink is consumed is indicated in each item.

**Table 3:** Psychology and body satisfaction data.

					95% Confidence Interval of the Difference	
Variable	Male	Female	T	*p*	Lower	Upper
Extraversion (Big Five)	5.6 ± 1.7	4.9 ± 1.6	4.401	0.000	0.36435	0.95161
Pleasant (Big five)	5.7 ± 1.8	5.9 ± 1.9	1.321	0.187	−0.53582	0.10489
Scrupulous (Big five)	6.4 ± 1.8	6.8 ± 2.1	2.353	0.019	−0.74059	−0.06682
Neuroticism (Big five)	4.9 ± 1.9	5.9 ± 2.4	5.462	0.000	−1.36750	−0.64420
Openness to Experience (Big five)	6.5 ± 2.0	6.9 ± 2.2	2.173	0.030	−0.75389	−0.03813
ZUNG Score	47.4 ± 5.4	49.1 ± 5.5	3.504	0.000	−2.56506	−0.72262
AAQ II	20.7 ± 8.5	21.9 ± 9.5	1.624	0.105	−2.74040	0.26002
UCLA	4.3 ± 1.5	4.2 ± 1.7	1.092	0.275	−0.11971	0.41959
STAI	11.3 ± 3.4	11.4 ± 4.0	−3.473	0.001	−1.69633	−0.47094
Body Satisfaction (EDI)	18.2 ± 3.5	17.9 ± 3.5	1.113	0.266	−0.25846	0.93515

AAQ II (Acceptance and Action Questionnaire II); UCLA (UCLA Loneliness Scale); STAI (Spielberger State—Trait Anxiety Inventory); ZUNG (ZUNG Depression Scale).

**Table 4:** Health and physical activity data.

					95% Confidence Interval of the Difference	
Variable	Male	Female	T	*p*	Lower	Upper
Days you have been injured in the last year	5.2 ± 6.0	3.8 ± 4.1	1.205	0.229	−1.08069	4.51267
Number of training sessions per week	4.9 ± 19.9	3.1 ± 5.6	0.248	0.804	−2.74999	3.54523
Average time in minutes of weekly training	25.4 ± 13.2	17.8 ± 25.9	0.563	0.574	−22.84060	12.66806
Minutes of weekly aerobic training	11.7 ± 100.2	9.8 ± 113.3	0.501	0.616	−40.87648	24.25635
Bench press PR (kg)	62.3 ± 201.7	40.6 ± 173.4	0.156	0.876	−14.22743	12.12950
Back squat PR (Kg)	45.0 ± 44.3	29.7 ± 36.1	0.828	0.408	−16.05149	6.54848
Percentage of the week below fifty percent of the maximum load	21.7 ± 40.4	30.5 ± 47.4	1.784	0.075	−18.39401	0.89632
Percentage of the week between fifty and seventy percent of the maximum load	12.7 ± 18.9	21.7 ± 0.9	1.506	0.133	−20.57099	2.72550
Percentage of week between seventy and eighty-five percent of maximum load	9.6 ± 16.7	9.4 ± 18.2	0.151	0.880	−2.80138	3.26868
Percentage of training of week conducted above eighty-five percent	10.4 ± 19.0	13.1 ± 19.3	1.471	0.142	−7.66770	1.10683
Smoking	2.3 ± 0.8	2.2 ± 0.8	0.980	0.327	−0.07190	0.21525
Experience frequent gastritis or heartburn	1.9 ± 0.5	1.9 ± 0.7	0.812	0.417	−0.06238	0.15024
Frequent dry throat sensation	2.0 ± 0.6	1.9 ± 0.7	2.604	0.009	0.03777	0.26943
Frequent dental sensitivity	2.1 ± 0.6	1.9 ± 0.8	1.961	0.050	−0.00017	0.23821
Days sick throughout the year	3.6 ± 7.8	2.3 ± 3.4	2.388	0.017	0.24277	2.49120

The smoking frequency is daily.

## Data Availability

Data are contained within the article.
